# A Cumulative Effect by Multiple-Gene Knockout Strategy Leads to a Significant Increase in the Production of Sophorolipids in *Starmerella Bombicola* CGMCC 1576

**DOI:** 10.3389/fbioe.2022.818445

**Published:** 2022-03-09

**Authors:** Jun Liu, Xinyu Zhang, Guodong Liu, Guoqin Zhao, Xiaoran Fang, Xin Song

**Affiliations:** ^1^ State Key Laboratory of Microbial Technology, Shandong University, Qingdao, China; ^2^ National Glycoengineering Research Center, Shandong University, Qingdao, China

**Keywords:** starmerella bombicola, high-yield, sophorolipids, rim9-like protein, transcription factors

## Abstract

Sophorolipids (SLs), an important biosurfactant produced by *S. bombicola*, were one of the most potential substitutes for chemical surfactants. Few reports on the transcriptional regulation of SLs synthesis and the engineered strains with high-yield SLs were available. In this study, a Rim9-like protein (Rlp) and three transcription factors (*ztf1*, *leu3*, *gcl*) were mined and analyzed, and a progressive enhancement of SLs production was achieved through cumulative knockouts of three genes. The sophorolipid production of *ΔrlpΔleu3Δztf1* reached 97.44 g/L, increased by 50.51% than that of the wild-type strain. Compared with the wild-type strain, the flow of glucose to SLs synthesis pathways was increased, and the synthesis of branched-chain amino acids was reduced in *ΔrlpΔleu3Δztf1*. The amount of UDP-glucose, the substrate for two glycosyltransferases, also increased, and the expression level of the key genes *sble* and *UGPase* for SLs synthesis increased by 2.2 times, respectively. The multiple-gene knockout strategy was proved to be highly effective to construct the engineered strain with high-yield SLs production, and this strain was a superior strain for industrial fermentation of SLs and reduced SLs production costs.

## Introduction

Surfactants are amphipathic compounds with both hydrophilic and hydrophobic moieties and have a wide range of applications. Most surfactants in use today are petroleum-based and chemically synthesized. However, such chemically-synthesized surfactants are difficult to degrade through the action of microorganisms (Santos et al., 2016), and thus cause significant ecological problems, particularly in washing applications as these surfactants inevitably end up in the environment after use. Biosurfactants have several advantages over their chemically produced counterparts. Such advantages involve non-toxicity, biodegradability, bioavailability, biocompatibility, eco-friendliness, effectiveness and stability at extreme environments (active in broad range of pH, temperature and salt concentrations), and long storage time. In addition, because of their antimicrobial, antiviral, anti-tumor and other biological activities, biosurfactants will have great application potential in the fields of clinical therapy and pharmacy (Anjum et al., 2016; Gaur et al., 2019; Valotteau et al., 2019). Therefore, biosurfactants attract increasing interests from researchers.

Sophorolipids (SLs) are one of the most studied biosurfactants, which are mainly produced by certain species of nonpathogenic yeasts. They can be used as a food emulsifier and antibacterial agent (Gaur et al., 2019), or as a coating for curcumin or lutein to enhance water solubility and bioavailability (Peng et al., 2018; Yuan et al., 2019). SLs are produced as a mixture of more than 20 different sophorolipid molecules. Each sophorolipid molecule is composed of hydrophilic sophorose molecules connected by a glycosidic bond with a hydrophobic long-chain hydroxy fatty acid. Some small modifications occurred on the basic chemical structures produce different sophorolipid molecules, these small modifications include acetylation or not and the acetylation position, the number of acetyl groups on the sophorose head, different carbon chain length and saturation degree on hydroxy fatty acid moieties. The key enzymes of SLs synthesis pathway have been revealed to be grouped in one large subtelomeric gene cluster (Van Bogaert et al., 2013; Anjum et al., 2016). First, fatty acids are converted into the ω-/ω-1 hydroxy fatty acids by the catalysis of cytochrome P450 monooxygenase Cyp52m1. Then, UDP-glucose is coupled with the hydroxyl group of fatty acid (position C1’), and the glycolipid is generated under the action of glucosyltransferase I (Ugta1). Next, the second UDP-glucose is coupled to the C2’ position of the first glucose moiety through glycosyltransferase II (Ugtb1) to form a non-acetylated acid SLs. It is further modified by acetyl transferase (Slat) and lactone esterase (Sble) to obtain acetylated acidic or lactonic SLs (Roelants et al., 2014; Ciesielska et al., 2016).

Due to the highest yield of all the biosurfactants, SLs are considered the most likely alternatives to chemical surfactants. However, the high production cost of SLs still makes them uncompetitive with chemical surfactants, which is an obstacle to their large-scale commercialization. At present, the research on SLs mainly focused on the mining and functional analysis of the enzymes related to SLs synthesis, fermentation optimization of SLs production and utilization of cheap fermentation substrates, including biomass raw materials to achieve sustainable production of SLs, and application expansion of SLs (Gaur et al., 2019; Kim et al., 2021; Li J.-f et al., 2021). There have been only a few reports of the genetic engineering of SLs-producing strains to increase the yield of SLs (Van Bogaert et al., 2009), and few studies on transcriptional regulators involved in the biosynthesis of SLs were available. The lactone esterase Sble that catalyzes the formation of a closed ring of acetylated acidic SLs was identified by analyzing the extracellular proteome of *S. bombicola* (Ciesielska et al., 2016). Yang et al. used the MFE-2 mutant strain to reveal the key role of acetyl-CoA in the synthesis of SLs through metabolic profiling and flux distributions. The supplementation of citric acid restored the yield of SLs by approximately 83% (Yang et al., 2019). Lodens et al. revealed the potential regulation of SLs synthesis by the telomere positioning effect, as the SL gene cluster of *S. bombicola* is located adjacent to a telomere. When a second SLs synthesis gene cluster was introduced elsewhere of the genome and was far away from the telomere, the synthesis of SLs in the mutant strain was shifted from the stationary phase to the exponential phase strict regulation of SLs synthesis in the stationary phase of *S. bombicola* (Lodens et al., 2020).

The purpose of this study was to identify transcription factors (TFs) and genes associated to the biosynthesis of SLs by analyzing the genome data of *S. bombicola*, and to unravel the mechanism that affects the synthesis of SLs, and finally to construct high SLs-producing strains through the combination of positive promoting factors.

## Materials and Methods

### Strain and Culture Conditions

The yeast strain, *S. bombicola* CGMCC 1576 was isolated from an oil-containing wastewater sample by our laboratory and preserved in China General Microbiological Culture Collection Center (CGMCC). The strain *S. bombicola* CGMCC 1576 was used as a wild-type strain in this study. Acetonitrile and methanol were of chromatographic grade and purchased from TEDIA Company Inc. (Fairfield, United States). Anthrone was of chemical grade and purchased from Sigma (St. Louis, United States). Agar powder, galactose, sorbitol, and other reagents were of chemical grade and purchased from Dingguo (Beijing, China).

The yeast strains were cultivated in YPD medium containing (per liter) 10.0 g of yeast extract, 20.0 g of peptone and 20.0 g of glucose (galactose-induced medium in which glucose was replaced by galactose). The SC (yeast synthetic medium) contained (per liter): yeast nitrogen base 1.7 g (NH_4_)_2_SO_4_ 5.0 g, yeast synthetic drop-out medium supplements 1.3 g, galactose 20.0 g, for induced deletion of *hph* in mutants. The solid medium was supplemented with 2.0% agar. The fermentation medium contained (per liter): glucose 80.0 g, oleic acid 60.0 g, yeast extract 3.0 g, KH_2_PO_4_ 1.0 g, Na_2_HPO_4_.12H_2_O 1.0 g, and MgSO_4_.7H_2_O 0.5 g, unless otherwise specified, this medium was used as fermentation medium, for SLs production.

### Strain Construction

The whole genome of *S. bombicola* CGMCC 1576 has been sequenced, the IPRO number of the sequence annotation of *S. bombicola* were compared with the IPRO number of the fungal transcription factor family, the possible transcription factors were screened, and the deletion strains of the transcription factor gene was constructed. Mutant strains were constructed by homologous recombination. Primers were designed to meet the following criteria: GC content 45–60%, Tm 50–60°C, and a length of 24 base pairs. The lengths of the 5 and 3′ flanking regions ranged from 1.0 to 1.5 kb for each gene. The chimeric primers for the amplification of upstream and downstream flanking fragments carried 25 bases of homologous sequence overlapping with the ends of *hph* marker sequence (5.2 kb). A final fragment of approximately 8.0 kb that contains target gene flanking sequences surrounding *hph* was created by double joint PCR and transformed into the wild-type *S. bombicola* by electroporation. The positive transformants of deletion mutant were singled out and purified on YEPD agar plates containing hygromycin B of a final concentration of 500 μg/ml. The resulting strains were verified by diagnostic PCR using primer pairs.

Multiple knockout strains were also constructed using hygromycin selection marker. Hygromycin selection marker was removed from single gene knockout strains in galactose-induced medium. First, single gene knockout strains were cultured in induction medium overnight, and then streaked on YPD plates to grow for 4 days. Single colonies were picked on YPD solid plates with and without hygromycin, and cultured in a constant temperature incubator at 30°C. A single colony that could grow on a plate without hygromycin and could not grow on a plate with hygromycin was picked and inoculated in a test tube of liquid YPD medium. The genome of the transformant was extracted to verify whether the hygromycin gene had been deleted. The correct transformant was used as the starting strain, then the knockout cassette of the second gene was transferred to the transformant with single gene knocked out and then a double genes knockout strain was obtained. Primers used to construct the strain were supplemented in the Supplementary Figure S1.

### Fermentation

The knockout strains and wild-type strains stored in glycerol tubes were activated in liquid YPD medium in test tubes, 2% (vol/vol) of yeast suspension was inoculated into liquid YPD medium in test tubes and cultured overnight until OD_600_ = 1.0, then 2% (vol/vol) of culture broth was transferred into 50 ml fermentation medium in 300 ml shake flasks, cultured at 30°C, 200 rpm for 7 days. All fermentations were performed in triplicate.

### Analytical Methods

After fermentation, the parameters of the fermentation broth were analyzed as described in our previous work (Liu et al., 2020). Residual glucose content was measured by a biosensor SBA-40C (Shandong Academy of Sciences, Jinan, China). For determination of cell dry weight, one volume of fermentation broth was added to five volumes of n-butanol/ethanol/chloroform (10:10:1) solution. The mixture was shaken well, after standing, then centrifuged at 8,000 rpm for 10 min. The supernatant was removed and the pellet was washed twice with distilled water and then dried to a constant weight at 50°C.

The titer of SLs was measured using methods based on anthrone-sulfuric acid and further verified by gravimetric measurements (Li et al., 2016b). Two volumes of ethyl acetate were added to 0.5 ml of fermentation broth, and the solution was centrifuged at 10,000 rpm for 10 min. The lactonic SLs in organic phase were measured by anthrone method (Ma et al., 2011). For the determination of total SLs, 1 ml of ethyl alcohol was added to 0.5 ml of fermentation broth and the mixture containing total SLs and residual glucose was centrifuged at 10,000 rpm for 10 min, total sugar content in the supernatant was quantified by anthrone method. The total SLs production was calculated by subtracting the amount of residual glucose from the total glucose content. Analysis of SLs components was performed by high performance liquid chromatography (Li et al., 2016a).

### qRT-PCR and Transcriptome Analysis

Mutants and wild-type strains were grown in fermentation medium at 30°C, 200 rpm for 72 h. The fermentation broth was centrifuged at 4°C for 5,000 rpm, and the cells were collected for RNA extraction. The sample was ground into fine powder in liquid nitrogen, and total RNAs were extracted with RNAiso Plus reagent (TaKaRa, Japan). The cDNAs were synthesized using PrimeScript^TM^RT Reagent Kit with gDNA Eraser (TaKaRa, Japan). The transcription levels of the genes for SLs synthesis by using LightCycler^®^ 480 system (Roche, Germany) and SYBR^®^ Premix Ex Taq™ (Prefect Real Time) (TaKaRa, Japan). The filament gene actin (GenBank ID: KT002360) was used as the internal reference gene to calculate the relative fold change of transcription of sample genes.

The analysis of transcriptomics was based on the Illumina HiSeq sequencing platform, using Next-Generation Sequencing (NGS). The integrity of total RNA was detected by Agilent 2,100 Bioanalyzer, mRNA was purified through the unique poly structure of RNA. Using ion interruption, mRNA was interrupted to 200-300bp fragments. A 6-base random primer and reverse transcriptase were used to synthesize the first strand of cDNA by using RNA as a template, and the first strand cDNA was used as a template to synthesize the second strand cDNA. Finally, the sequencing work was completed by Shanghai Personal Biotechnology Co., Ltd (Shanghai, China). The raw data of each sample was counted separately, and some reads with connectors and low quality were filtered. Sequences with adapters at the 3′ end were removed using Cutadapt. Reads with an average quality score below Q20 were also removed. The filtered reads were aligned to the reference genome using HISAT2 (http://ccb.jhu.edu/software/hisat2/index.shtml) software. The Read Count value of the gene counted by HTSeq was used as the original expression level of the gene. The expression level was normalized by FPKM (Fragments Per Kilo bases per Million fragments). DESeq performs differential analysis on gene expression, and the conditions for screening differentially expressed genes were as follows: expression difference fold |log2FoldChange| > 1and significant *p*-value < 0.05. In NCBI Databank, the genome sequence of *S. bombicola* was uploaded by other researchers (https://www.ncbi.nlm.nih.gov/genome/?term=Candida%20Bombicola) and the genome was used as reference genome for transcriptomics analysis. The whole genome of *S. bombicola* CGMCC 1576 (a patent strain of our laboratory) were sequenced a few years ago and the genes were numbered by sequencing company (BGI Genomics Co., Ltd.). Last year, the whole genome of *S. bombicola* CGMCC 1576 were resequenced and the data were uploaded to the NCBI databank. The link address is: https://www.ncbi.nlm.nih.gov/genome/36523?log$=activity.

### Nucleotide Sequence Accession Number

The GenBank accession number for genes were as follows: *rlp (gme3787)*, MZ047266, *ztf1* (*gme3596*), MZ047267, *leu3* (*gme 1908*), MZ047268, *gcl* (*gme5*), MZ047269. The raw data of expression profiling were deposited in NCBI’s Gene Expression Omnibus (GEO) database under the accession number GSE138083.

### Statistical Significance Tests

All statistical significance tests were done with a one-tailed homoscedastic (equal variance) *t*-test in Excel 2019. *p < 0.05, **p < 0.01, ***p < 0.001.

## Results

### Rlp Affects the Synthesis of SLs in *S. Bombicola*

**FIGURE 1:**
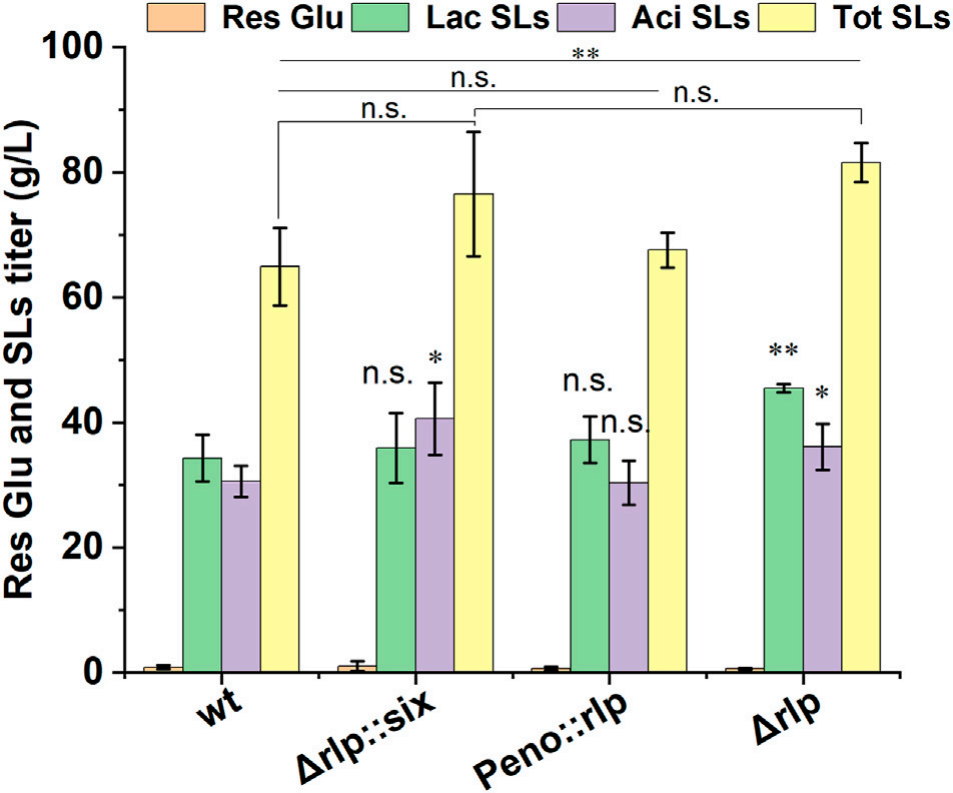
Phenotypic analyses of deletion and overexpression strain of *rlp* cultured on glucose and oleic acid. *wt*: wild-type strain, *Res Glu*: residual glucose, *Lac SLs*: lactonic SLs, *Tot SLs*: total SLs. Results were obtained from at least three biological replicates. Error bars represent standard deviation. Statistical analysis was performed using Student’s t-test (one-tailed; *p ≤ 0.05, **p ≤ 0.01, ***p ≤ 0.001, n. s: not significant; two-sample equal variance).

The pH value of fermentation medium not only affects the production of SLs, but also affects the components of SLs (Ma et al., 2011). In *S. cerevisiae*, the expression of alkaline pH-responsive genes was regulated by the transcription factor Rim101, and the proteolysis of Rim101 was activated (catalyzed) by Rim9, a plasma membrane protein (Gonzalez-Lopez et al., 2002). It is speculated that there is a homologous protein of rim9 in *S. bombicola*, which affects the synthesis of SLs by directly or indirectly affecting the expression of key genes for SLs synthesis. Rlp was identified in *S. bombicola* by analyzing and comparing genomic data with the amino sequence of Rim9 in *Y. lipolytica*. Rlp in *S. bombicola* shared 30.5% identities and 55.4% sequence similarity with Rim9 in *Y. lipolytica*. To further investigate the function and the effect of Rlp on the synthesis of SLs, the knockout strain (Δ*rlp::six*) and overexpression strain (*Peno::rlp*) of *rlp* were constructed in *S. bombicola* through homologous recombination. Wild-type strain, Δ*rlp::six* and *Peno::rlp* were incubated in fermentation medium at 30°C, 200 rpm for 7 days. Fermentation results were shown in Figure 1.

The titer of SLs in Δ*rlp::six* reached 76.5 g/L, which was increased by 17.9% compared with that of the wild-type strain. The amounts of lactonic SLs were equivalent to that of the wild-type strain, and the titer of acidic SLs increased by 32.7%. However, the titers of total SLs and lactonic SLs in *Peno::rlp* were the same as those of the wild-type strain, indicating that the overexpression of *rlp* did not affect SLs production in *S. bombicola*.

Given that the deletion of *rlp* can significantly enhance the SLs production ability of the strain, the knockout strain Δ*rlp::six* could be used as an original strain to further genetic manipulations to construct a high SLs-producing strain.

### Screening of TFs Related to SLs Synthesis in *S. Bombicola*

Researchers have done a lot of work to optimize the fermentation process of SLs (Ma et al., 2019; Yuning et al., 2019). However, there is little research on the regulation of SLs synthesis by TFs. In order to uncover the TFs involved in the regulation of SLs synthesis in *S. bombicola*, the fungal transcription factor database (http://ftfd.snu.ac.kr/tf.php) was compared with the whole genome sequencing data, and 158 possible TFs were found in *S. bombicola* CGMCC 1576. Ninety genes in several large TF families were knocked out using homologous recombination (These genes’ IDs were shown in Supplementary Figure S2). These knockout strains were fermented to produce SLs in 50 ml fermentation medium of 300 ml shake flasks. After preliminary screening, three negative regulators (*ztf1, leu3 and gcl*) of SL-synthesis were identified in *S. bombicola*, the results were shown in Table 1.

**TABLE 1:** Phenotypic analyses of TF deleted strains cultured on glucose and oleic acid.

Strain	Res glu (g/L)	Lac SLs (g/L)	Tot SLs (g/L)	Increase the lac SLs (%)	Increase the tot SLs (%)
wt	0.88 ± 0.35	34.30 ± 3.76	64.89 ± 6.25	0	0
*∆ztf1*	7.57 ± 4.27	26.79 ± 3.97	72.13 ± 10.00	−21.89	11.15
*Δgcl*	0.48 ± 0.04	32.93 ± 5.01	74.75 ± 4.49	−3.99	15.19
*Δleu3*	1.40 ± 0.09	30.42 ± 4.79	76.85 ± 5.21	−11.32	18.43

*Wt*: wild-type strain, *Res Glu*: residual glucose, *Lac SLs*: lactonic SLs, *Tot SLs*: total SLs.

Compared with the wild-type strain, the total SLs production of *Δztf1*, *Δleu3* and *Δgcl* reached 72.13 g/L, 76.85 g/L and 74.75 g/L, increased by 11.15, 18.43 and 15.19%, respectively. The titers of lactonic SLs were 26.79 g/L, 30.42 g/L and 32.93 g/L, respectively. Compared with the wild-type strain, the lactonic SLs of these three knock-out strains were all reduced, the lactonic SLs of *Δztf1* decreased the most, by 21.89%. The production of acidic SLs of these three knock-out strains all increased, and the acidic SLs of *Δleu*3 increased the most, by 51.80%, when compared with that of the wild-type strain.

The online analysis tool Blast P (http://blast.ncbi.nlm.nih.gov/Blast.cgi) was used for sequence alignment and analysis. Through online comparison and analysis of the protein sequences of TFs, it was found that Leu3 (*gme 1908*) in *S. bombicola* shared 28% identity of amino acid sequence with the Leu3 zinc-knuckle transcription factor in *S. cerevisiae*. TF Leu3 in *S. cerevisiae* regulates genes involved in branched chain amino acid biosynthesis and ammonia assimilation (Friden & Schimmel, 1987). A GME5 protein in *S. bombicola* shared 26% identity with Gcn4 in *S. cerevisiae*, a bZIP transcriptional activator of amino acid biosynthetic genes, which responds to amino acid starvation in *S. cerevisiae* (Natarajan et al., 2001). However, when the amino acid sequence of Gcn4 protein in *S. cerevisiae* was subjected to genome data of *S. bombicola* blast analysis, the protein GME483 was detected in *S. bombicola*, which showed a higher level of sequence identity (34%) with the Gcn protein in *S. cerevisiae*. Based on the above analysis and blast, GME5 was named as Gcn4-like protein (Gcl). The protein GME3596 belongs to the family of zinc-knuckle transcription factor, no homologous protein has been reported, and thus the GME3596 protein in *S. bombicola* was named as Ztf1 (zinc-knuckle transcription factor 1).

### Genetic Engineering to Improve SLs Production

Knockout of 1 TR has been shown to increase the titer of SLs. It is speculated that multiple knockouts of TFs can continuously increase the SLs production of mutant strains. Therefore, *Δrlp::six* was selected as the initial strain for further knockout manipulations of two and 3 TFs.

First, galactose was used to induce deletion of the *hph* expression cassette in *Δrlp::six*. *Δrlp::six* strains were cultured in a medium using galactose as a carbon source overnight, and were picked for streaking, spotting and counter-screening. Six knockout strains were selected and the hygromycin expression cassette was verified to be deleted. *Δrlp::six* and Δ*rlp* were used to produce SLs, and the effect of *hph* self-deletion on the mutants was analyzed. As shown in Figure 1, compared with *Δrlp::six*, the SLs titer of the knockout strain with the hygromycin resistance expression cassette deleted did not change obviously, the titer of total SLs and lactonic SLs were 81.57 g/L and 45.44 g/L, respectively. The above data indicated that the deletion of the hygromycin resistance gene has basically no effect on the SL production of *Δrlp*.

**FIGURE 2:**
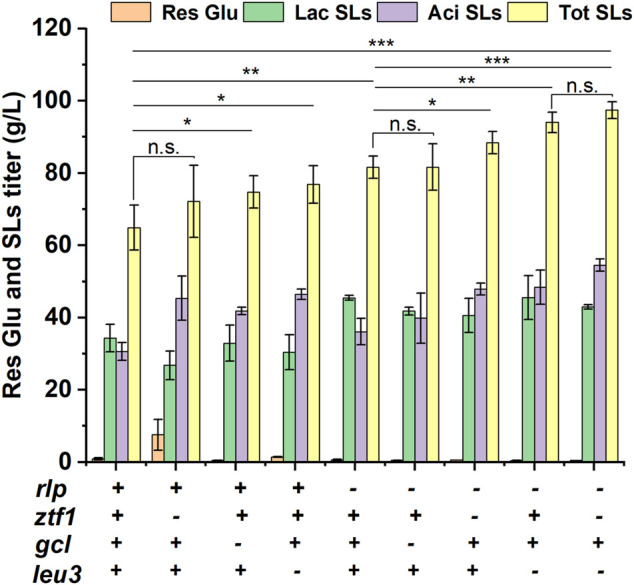
Phenotypic analyses of high-producing SLs strains. *Res Glu*: residual glucose, *Lac SLs*: lactonic SLs, *Tot SLs*: total SLs. “– “and “+ “symbols indicate absence or presence of the corresponding genetic change, respectively. Results were obtained from at least three biological replicates. Error bars represent standard deviation. Statistical analysis was performed using Student’s *t*-test (one-tailed; *p ≤ 0.05, **p ≤ 0.01, ***p ≤ 0.001, n. s: not significant; two-sample equal variance).

Then, 3 TFs related to SLs synthesis were deleted on the base of *Δrlp*, respectively and double-knockout strains *ΔrlpΔztf1*, *ΔrlpΔleu3* and *ΔrlpΔgcl* were obtained, respectively. These double knockout mutants were subjected to fermentation experiment and the results were shown in Figure 2 and Supplementary Figure S1. The titer of *ΔrlpΔztf1* and *ΔrlpΔleu3* SLs were significantly increased compared with the initial strain *Δrlp*. The total SLs of *ΔrlpΔztf1* reached 88.44 g/L, which was 8.42% higher than that of *Δrlp*, and the acidic SLs was increased by 32.45%. *ΔrlpΔleu3* produced 93.99 g/L of total SLs, which is the highest in these double knockout strains, the total SLs by *ΔrlpΔleu3* was improved by 15.22%, and the titer of acidic SLs increased by 34.05% compared to that by *Δrlp*. In the double knockout strain *ΔrlpΔgcl*, the fermentation performance of *ΔrlpΔgcl* was equivalent to that of *Δrlp*, the total SLs production of *ΔrlpΔgcl* did not change, and the mean value of total SLs production was the same as that of *Δrlp*, the lactonic SLs decreased significantly (from 45.44 g/L to 41.78 g/L), and the acidic SLs increased only modestly, from 36.12 g/L to 39.84 g/L. However, the lactonic SLs production of *ΔrlpΔztf1* and *ΔrlpΔleu3* did not change compared with that of *Δrlp*, while the titer of acidic SLs was significantly increased, increasing by 32.47 and 34.09%, respectively. We speculated that the mechanism of *ztf1* and *leu3* regulating SLs synthesis was probably different from that of *rlp*, so the further knockout of *ztf1* or *leu3* in *Δrlp* showed superimposed effects on total SLs synthesis. The double knockout strain *ΔrlpΔgcl* which was constructed by deleting *gcl* in *Δrlp* showed no accumulation effect on SLs synthesis compared to *Δrlp*.

In addition, in order to continue to increase SLs production, the double knockout strain *ΔrlpΔleu3* with the highest titer was selected as the starting strain of three-gene knockout strain, *ztf1* was knocked out in *ΔrlpΔleu3*, and a knockout strain (*ΔrlpΔleu3Δztf1*) with three genes knocked out was obtained. The results were shown in Figure 2. The titer of total SLs was 97.44 g/L in *ΔrlpΔleu3Δztf1*, which was increased by 3.67% compared to that of *ΔrlpΔleu3*, and the carbon source conversion efficiency yield (g/g) which represents the ratio of the yield of SLs to the total amount of added substrate (glucose and oleic acid) was 0.70. The titer of total SLs in *ΔrlpΔleu3Δztf1* increased by 50.15%, and lactonic SLs increased by 25.25%.

The main SL molecules produced by wild-type strain, *Δrlp*, *ΔrlpΔleu3* and *ΔrlpΔleu3Δztf1* were analyzed by HPLC, as shown in Supplementary Figure S2 and Supplementary Figure S3. Compared with that in the wild-type strain, the peak 3 and peak 5 were increased in *ΔrlpΔleu3Δztf1*, respectively. According to the results of our previous work (Li et al., 2016a), peak 3 and peak 5 were identified as C18:2 DASL and C18:1 DLSL. C18:2 DASL has excellent surface activity, and C18:1 DLSL has been reported to have the strongest biological activities including a strong inhibitory effect on human esophageal cancer cells, KYAE 109 and KYSE 450 (Shao et al., 2012), which makes the strain *ΔrlpΔleu3Δztf1* be a high producer of C18:1 DLSL.

### Analysis of Regulation Mechanism of Two TFs and Rlp on SLs Synthesis

**FIGURE 3:**
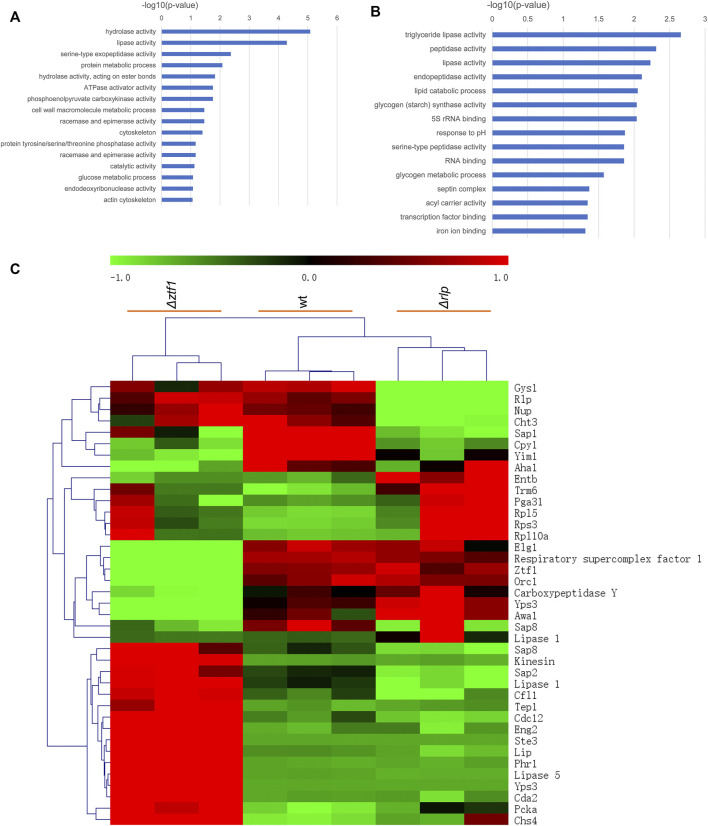
GO enrichment analysis and heatmap results. **(A)** Go enrichment analysis of *Δztf1*. **(B)** Go enrichment analysis of *Δrlp*. **(C)** Heatmap result in wt, *Δztf1* and *Δrlp*.

**FIGURE 4:**
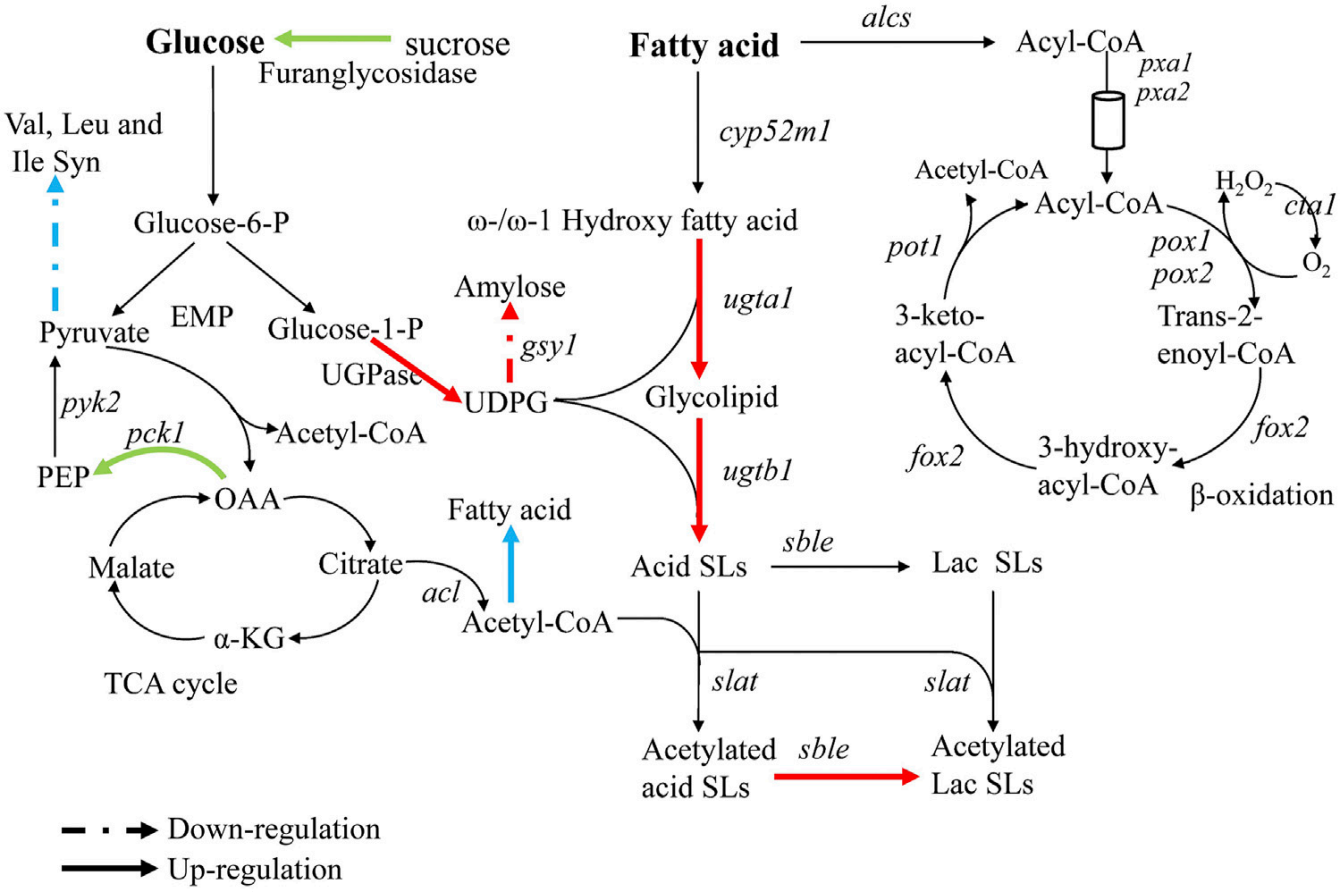
Analysis of transcriptional changes in *Δrlp*, *Δleu3*, and *Δztf1*. The blue arrow: *Δleu3*, green arrow: *Δztf1*, red arrow: *Δrlp*. Dotted lines indicate down-regulation of gene expression, solid lines indicate up-regulation.

**FIGURE 5:**
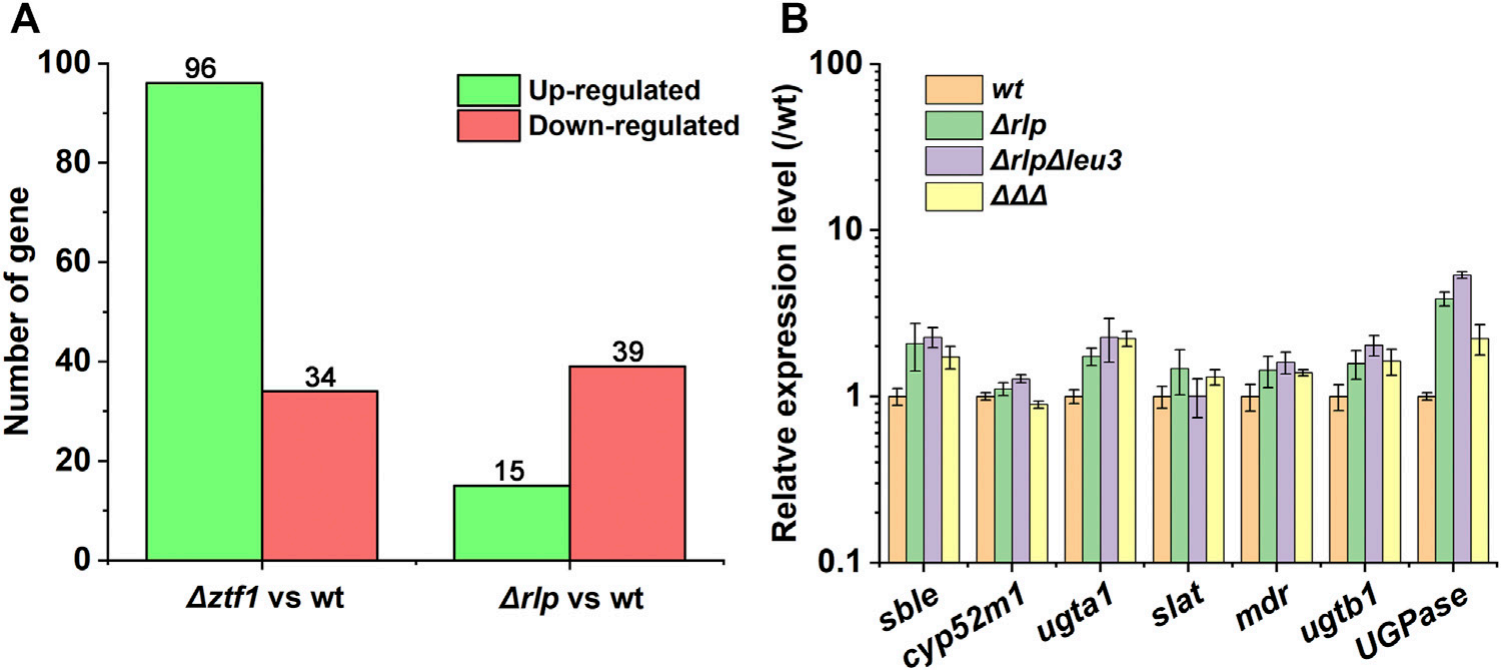
Expression difference analysis result. **(A)** Expression difference analysis result statistics. The conditions for screening differentially expressed genes are: the multiple of expression difference |log2FoldChange| > 1, and the significance *p*-value < 0.05. **(B)** The different transcriptional levels of the key genes for SLs synthesis in *Δrlp*, *ΔrlpΔleu3*, *ΔrlpΔleu3Δztf1* and wild-type strain. *ΔΔΔ*: *ΔrlpΔleu3Δztf1*.

In order to explain the reasons for the cumulative production of SLs in the triple knockout strains *ΔrlpΔleu3Δztf1*, and explore the regulation mechanism of 2 TFs Leu3, Ztf1, transcription level of the key genes for the SLs synthesis were analyzed by qRT-PCR. We also performed the whole-genome transcriptome profile of *Δztf1*, *Δrlp* and wild-type strains during fermentation processes using NGS based on the Illumina HiSeq sequencing platform (As shown in Figures 3–5). *Δleu3* in Figure 4 was based on qRT-PCR data, the other two mutants and Figure 5A were based on RNAseq, Figure 5B was based on qRT-PCR.

The whole genome data of *S. bombicola* was served as the reference genome data. First, the raw data was filtered, and the filtered high-quality sequence (Clean Data) was compared to the reference genome of *S. bombicola*. The samples were further analyzed for expression difference analysis, enrichment analysis and cluster analysis by calculating the expression level of each gene.

*Δztf1* exhibited 130 differentially expression genes (DEGs), there were 96 genes upregulated and 34 down-regulated. Then, *Δrlp* exhibited distinct expression of 54 genes. Among them, 39 genes were down-regulated and 15 genes were up-regulated compared to wild-type strains. To analyze the whole-genome transcriptional changes, GO term enrichment analysis and heatmap results of differentially expressed genes were performed (Figure 3). Lipase activity was significantly enriched in *Δztf1* and *Δrlp* at the same time. In addition, hydrolase activity, phosphoenolpyruvate carboxykinase activity and glucose metabolic process were enriched in *Δztf1*. Similarly, in the single knockout strain *Δrlp*, lipid catabolic process and glycogen metabolic process were enriched.

To analyze the reason causing cumulative effect in the double knockout mutants, the gene expression involved in SLs synthesis-related pathways were examined (The results were shown in Figure 4). The expression level of phosphoenolpyruvate carboxykinase (*pck1*) in *Δztf1* was up-regulated by 2.3 times. Oxaloacetic acid was catalyzed to form phosphoenolpyruvate by Pck1, which was the precursor material for the synthesis of pyruvate. Acetyl-CoA and ATP produced from pyruvate were essential for the synthesis of SLs. Acetyl-CoA was the substrate for the acetylation of non-acetylated SLs. Secondly, ATP could provide the energy for the entire metabolic process of SLs synthesis. In addition, the expression of β-fructofuranosidase was also up-regulated by 2.3 times. β-fructofuranosidase could promote the metabolic conversion of sucrose or starch to glucose, which makes more glucose flow to SLs synthesis. These two enzymes, Pck1 and β-fructofuranosidase promoted the metabolism of carbon and increased the flux of carbon to the synthetic pathway of SLs, respectively, thereby promoting the synthesis of SLs in *Δztf1*, but did not directly act on the key genes for SLs synthesis.

Unlike in *Δztf1*, the expression of glycogen synthase (*gsy1*) was down-regulated by 0.5 times in *Δrlp*. Amylose was synthesized by Gsy1 using UDP-glucose as a substrate, and UDP-glucose was a key metabolic intermediate in the synthesis pathway of SLs. Thus, the down-regulated *gsy1* expression reduces the competitive consumption of UDP-glucose in another metabolic pathway. In addition, through analyzing the results of qRT-PCR (Figure 5B), the expression levels of the key genes *sble* and *UGPase* for SLs synthesis in *Δrlp* were 2.1 and 3.9 times higher than those of the wild-type strain. These results indicated that deletion of *rlp* not only reduces the synthesis of polysaccharides from UDP-glucose but also increases the high expression of the key enzyme genes for SLs synthesis to a certain extent. The above results demonstrated that *ztf1* and *rlp* affect SLs synthesis through different metabolic pathways.

According to the results of bioinformation, Leu3 belongs to Zinc-knuckle transcription factor, repressor, and activator, which regulates genes involved in branched-chain amino acid biosynthesis. The transcription level of branched-chain amino acid transaminase (Bat1) was analyzed by qRT-PCR and found that the transcription amount of *bat1* in Δ*leu3* was down-regulated by 0.5 times compared to the wild-type strain, indicating that the deletion of *leu3* reduced the carbon flux to the synthesis of branched-chain amino acids, therefore, more glucose can be used for SLs synthesis in Δ*leu3*. Combination of diacylglycerol acyltransferase (Dga1) overexpression with nitrogen limitation resulted in a high level of lipid accumulation accompanied by downregulation of several amino acid biosynthetic pathways (including that of leucine in particular) in *Y. lipolytica* (Kerkhoven, 2017). Under the condition of limited nitrogen sources and both addition of hydrophobic and hydrophilic carbon sources (such as rapeseed oil and glucose), SLs are synthesized in large quantities after cells enter stationary phase (Rau et al., 1996). Large amounts of SLs occurred under nitrogen-limiting conditions, and after deleting the transcription factor *leu3*, the expression of DGA1 in *Δleu3* was up-regulated by 2.6 times compared with that in the wild-type strain. Therefore, we speculated that the lack of *leu3* promotes the fatty acid synthesis and then provide additional fatty acids for SLs synthesis, in the meantime reduces glucose to flow to the synthesis of branched-chain amino acids, thereby increased the production of SLs in *Δleu3*.

Next, the expression levels of SLs synthesis-related genes were analyzed by qRT-PCR in the wild-type strain, *Δrlp*, *ΔrlpΔleu3*, and *ΔrlpΔleu3Δztf1*, and the results were shown in Figure 5B. Compared with the wild-type strain, the expression levels of *sble*, *UGPase*, *ugta1*, and *ugtb1* in the three knockout strains were significantly increased. However, the expression levels of *cyp52m1*, *slat* and *mdr* did not changed significantly. Among them, the expression levels of the two glycosyltransferases *ugta1*, and *ugtb1* in *ΔrlpΔleu3Δztf1*, the two key genes for SLs synthesis, were both 2.2 times higher than that of the wild-type strain.

## Discussion

Claus et al. reported earlier this year, by screening 254 putative transporter genes, SbSLMdr.2 was found to be responsible for the transport of SLs in the absence of rapeseed oil (Claus et al., 2022). In this study, by screening the proteins and transcription factors that affect the synthesis of SLs, it was found for the first time that the deletion of *rlp*, *leu3*, and *ztf1* affected the synthesis of SLs. And multiple gene knockout resulted in a cumulative increase in SLs production of *S. bombicola* CGMCC 1576. Interestingly, the carbon source conversion rate of *ΔrlpΔleu3Δztf1* in the shake flask reached 0.70 (total carbon source added to the medium), which was higher than the optimized fermentation result of 0.68 (calculated based on the consumed carbon source, rather than the total amount of carbon source added). The volumetric productivity of SLs was 13.9 g L^−1^ d^−1^ in *ΔrlpΔleu3Δztf1*. It was lower than the optimized fed-batch result of 57 g L^−1^ d^−1^ (Rau et al., 2001). The productivity of SLs synthesis in *ΔrlpΔleu3Δztf1* was limited by insufficient substrate in the medium at the later stage of the fermentation in shaking flask. Compared with the wild-type strain, the up-regulated flow of glucose-1-P to UDP-glucose, OAA to PEP and acetyl-CoA to fatty acid in *ΔrlpΔleu3Δztf1* will lead to a reasonable down-regulation of the flow in the TCA cycle and subsequent reduction in biomass, which may hinder productivity of mutant strains (Figure 4).

SLs production can be generally enhanced by the increase of the transcription levels of key genes of SLs synthesis*.* It was recently reported that *Vitreoscilla* hemoglobin expression raised the transcription level of key genes of SLs synthesis, also enhanced SLs production under oxygen limited conditions (Li Y et al., 2021). In the present study, compared with the wild-type strain, the expression level of key enzyme genes responsible for SLs synthesis was up-regulated in the multi-knockout strain (*ΔrlpΔleu3Δztf1*), which probably means that the upregulation of transcription level of key genes of SLs synthesis contributed most to the increase of SLs production by *ΔrlpΔleu3Δztf1*. However, most genes expression of key enzyme genes in SLs synthesis pathway in the multiple knockout strains were lower than that of the single deletion and double deletion strains, like *sble*, *cyp52m1* and *UGPase*. As *ztf1* was deleted, the expression of key genes for SLs synthesis in the strain was down-regulated (data were shown in Supplementary Figure S3). Therefore, in the triple deletion strain obtained by deleting *ztf1* in the double knockout strain, the expression level of key genes for SLs synthesis was lower than that of the double knockout and single knockout strains. Therefore, in the future, researchers can consider adjusting the expression of the SLs synthesis gene cluster on the basis of the three knockout strains to continue to increase the SLs production of *ΔrlpΔleu3Δztf1*.

This study revealed for the first time the transcription factors related to SLs synthesis, which provided a theoretical basis for the follow-up metabolic modification of strains. The high-yield SLs-producing strains of *ΔrlpΔleu3Δztf1* can be considered as engineered strains for the industrial production of SLs.

## Data Availability

The datasets presented in this study can be found in online repositories. The names of the repository/repositories and accession number(s) can be found below: https://www.ncbi.nlm.nih.gov/geo/, GSE138083, https://www.ncbi.nlm.nih.gov/genbank/, MZ047266, https://www.ncbi.nlm.nih.gov/genbank/, MZ047267, https://www.ncbi.nlm.nih.gov/genbank/, MZ047268, https://www.ncbi.nlm.nih.gov/genbank/, MZ047269.
