# Supplementary material for: A Cumulative Effect by Multiple-Gene Knockout Strategy Leads to a Significant Increase in the Production of Sophorolipids in Starmerella Bombicola CGMCC 1576
Source: Front Bioeng Biotechnol. 2022 Mar 9;10:818445. doi: 10.3389/fbioe.2022.818445 (PMC8959766; doi:10.3389/fbioe.2022.818445)
Supplement: Supplementary file 1 [file DataSheet1.pdf]

Supplementary Material

1 Supplementary Figures and Tables

1.1 Supplementary Figures

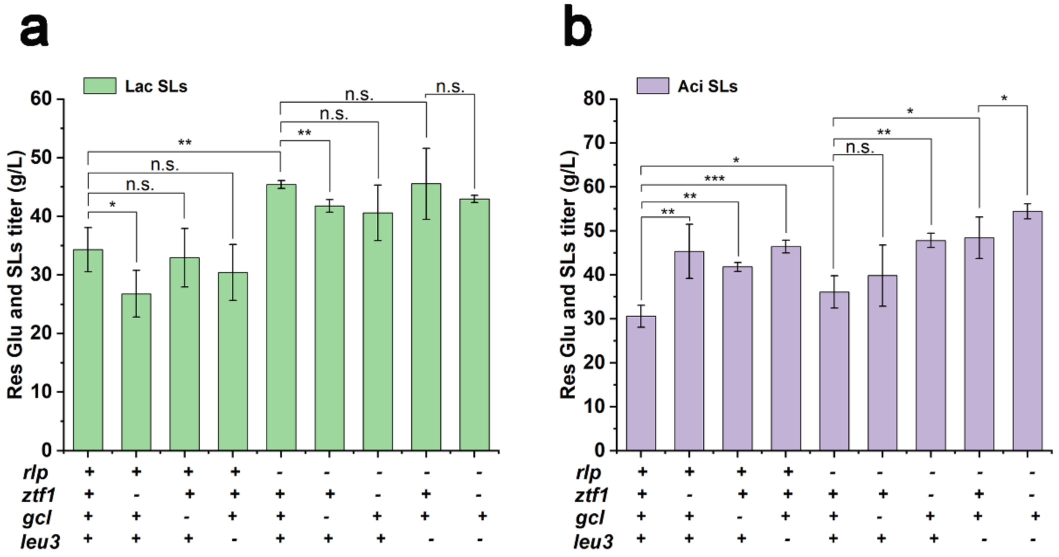

Supplementary Figure 1. Phenotypic analyses of high-producing SLs strains. *Lac SLs*: lactonic SLs, *Tot SLs*: total SLs. " – " and " + " symbols indicate absence or presence of the corresponding genetic change, respectively. Results were obtained from at least three biological replicates. Error bars represent standard deviation. Statistical analysis was performed using Student's t-test (one-tailed; * $P \leq 0.05$, ** $P \leq 0.01$, *** $P \leq 0.001$, n.s. : not significant; two-sample equal variance).

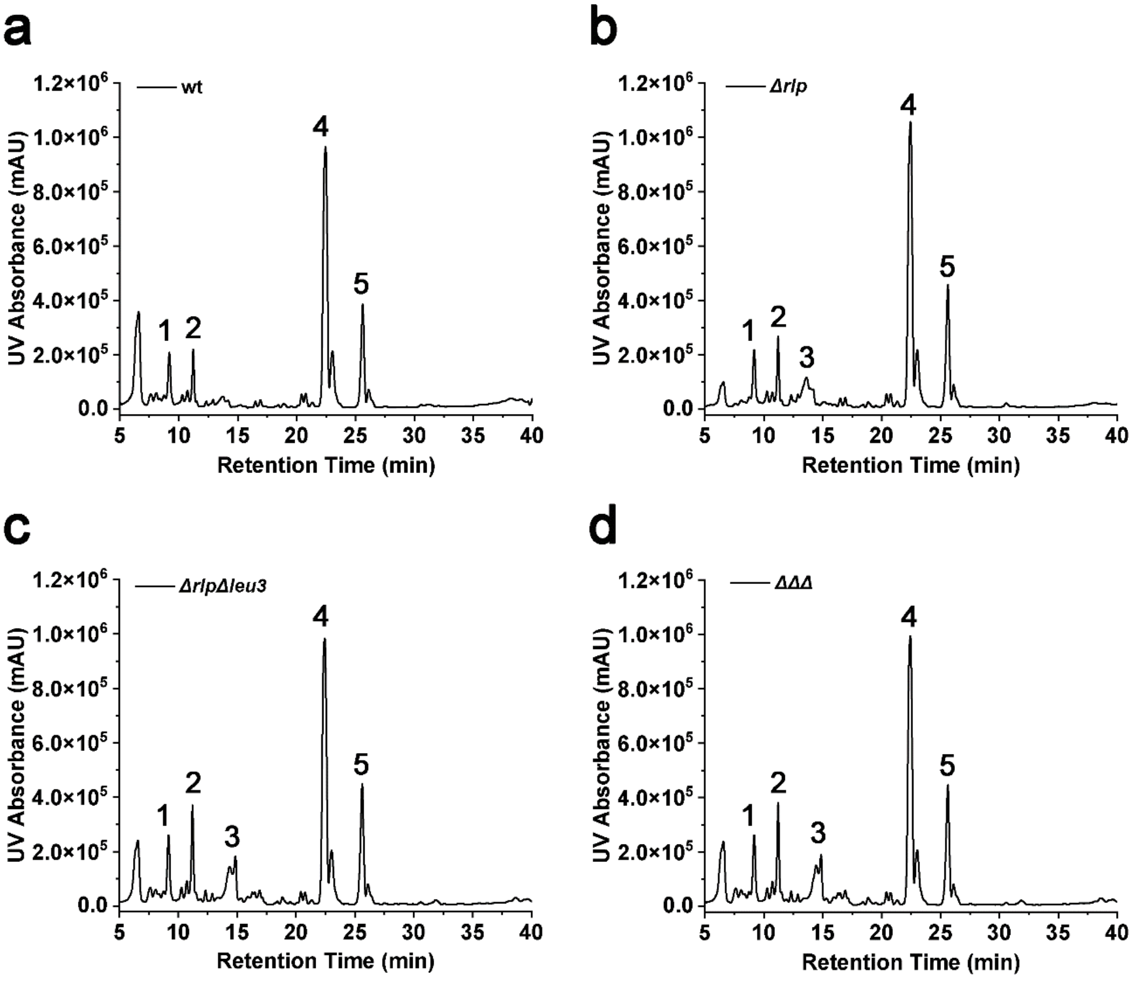

Supplementary Figure 2. The SLs' HPLC chromatogram. (1) C18:2 NASL, (2) C18:1 MASL, (3) C18:2 DASL, (4) C18:2 DLSL, (5) C18:1 DLSL, NASL non-acetylated acidic sophorolipid, MASL mono-acetylated acidic sophorolipid, DASL di-acetylated acidic sophorolipid, DLSL di-acetylated lactonic sophorolipid, wt: wild-type strain, ΔΔΔ: ΔrlpΔleu3Δztf1.

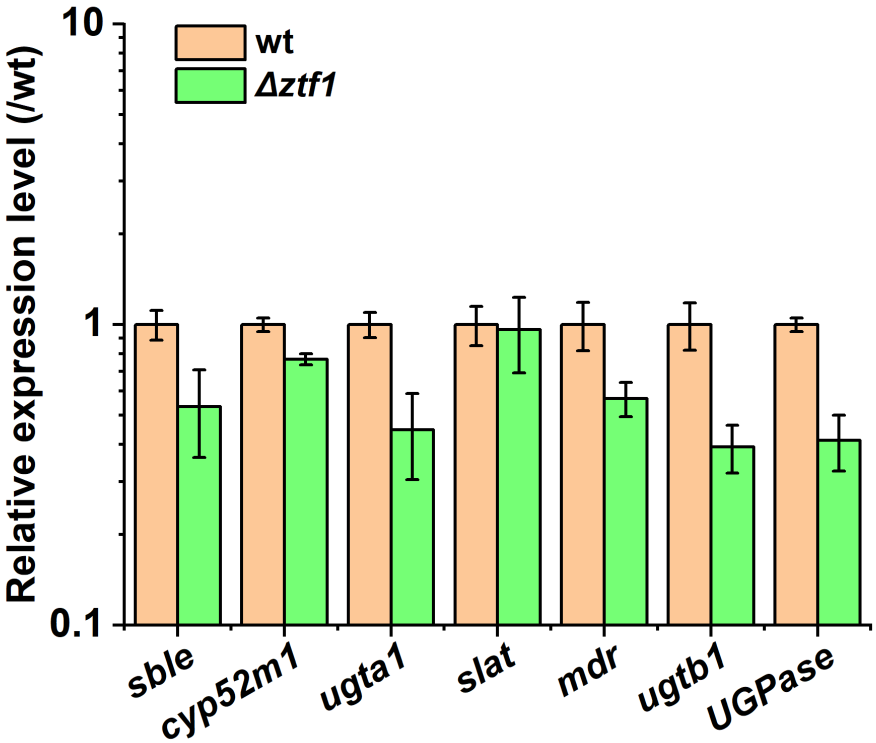

Supplementary Figure 3. The different transcriptional levels of the key genes for SLs synthesis in *Δztf1* and wild-type strain.

1.2 Supplementary Tables

Supplementary Table S1. Primers used for this study

Primers	Sequence (5' to 3')
Primers used in the construction of deletion mutants	
rlp-5'flankingfor	CTTGCCGAACTACGAGACTGG
rlp-5'flankingrev	CTGATGCGGTATTTTCTCCTTACGCCGAAGTCCTCTTTGGAGTGTTGTAG
rlp-3'flankingfor	AACATACGAGCCGGAAGCATAAAGTAGAGCCCTCTAATTTATGCTGCC
rlp-3'flankingrev	TGGACTGGTGAAGTTGCGTTTG
rec-hphfor	GCGTAAGGAGAAAATACCGCATCAG
rec-hphrev	ACTTTATGCTTCCGGCTCGTATGTT
rlp-gfor	ATGCTCTCACTGGAAACATTTACTG
rlp-grev	CAAATACTTGTGGAACTTGAATGAC
Primers used qRT-PCR	
cyp52m1-F	ATTGCCATTGAACTTCCGTA

cyp52m1-R	CCCCAATACTTGATGTCCCT
ugta1-F	GCCACTCTGTTCCAGTTGTTA
ugta1-R	CTGTAGGCAAGCCAGTTTTC
ugtb1-F	GGCGTCACTTCCTGATAGC
ugtb1-R	GCTTGTCTACCAACATTCCTC
slat-F	TCGTGACTGCGACTCATCCT
slat-R	CCATACATTATTCCCCACCTTG
mdr-F	GCCACAGAGCTAAGGCATACAC
mdr-R	GAATCGAAGCGGAGCAGTTA
actin-F	GTCATCTGCTCAACGAAGTGTAT
actin-R	ATGTCCTTCTGAGCGGTCTG
ugpase-F	CAAGAAGGTCAGCGAGTTCCAGAAG
ugpase-R	ACCAGTGATGACAACATTCTCCAAG
dga1-F	TCTTGCTAAACCAGGACACTTCG
dga1-R	GGTAAAACCGAGATACTTGGTCATA
bat1-F	GCCAAGGAAGGATTTGCTCA
bat1-R	TTGATTCTTGTGATTCCTTCCAG

Supplementary Table S2. Possible transcription factor in *S. bombicola*

Transcription Factor Family	Gene ID
Zn2Cys6	***gme29, gme810, gme1038, gme1536, gme1860, gme1908, gme1941, gme2393, gme2891,*** *gme3045,* ***gme3052, gme3092****, gme3105, gme4332, gme38,* ***gme3814***
GATA type zinc finger	***gme191, gme783, gme1677, gme2088, gme2322, gme2620, gme3168, gme3348***
C2H2 zinc finger	***gme114, gme30, gme296, gme531,*** *gme596,* ***gme751, gme971, gme1095, gme1162, gme1330, gme1582, gme1737,*** *gme1765,* ***gme1901, gme2262, gme2377, gme2383, gme2456, gme2686, gme2702, gme3008,gme3262, gme3470, gme3596, gme3685, gme3732, gme3854****, gme3908,* ***gme3983, gme3990, gme4151, gme4241,*** *gme4264, gme4287, gme4295,* ***gme4494****, gme4512*
bHLH	*gme1091,* ***gme3042, gme3601, gme4451***
bZIP	***gme5, gme483,*** *gme767,* ***gme1175,*** *gme1196, gme1290****, gme1556, gme1587,*** *gme1895,* ***gme2384, gme3889, gme4267***
Forkhead	***gme2141, gme4020***
Homeobox	*gme573,* ***gme3428, gme3492,*** *gme4326,* ***gme1415, gme2562***
MADS-box	*gme4240, gme4497*
Myb	***gme97, gme489, gme660, gme1310, gme1586****, gme2437,* ***gme2935, gme3740, gme3749, gme4170, gme4213,*** *gme4437, gme4441*
APSES	*gme715, gme1855, gme1998, gme3486, gme4461*
HMG	*gme397, gme493, gme725, gme2040, gme4097*
Heteromeric CCAAT factors	***gme2254, gme619, gme1212, gme1494,*** *gme2823, gme3558, gme3619*
TEA/ATTS	***gme673***
Winged helix repressor DNA-binding	***gme58****, gme401,* ***gme658, gme1482, gme1732****, gme1954,* ***gme2118, gme2163, gme2853, gme2860,*** *gme3163, gme3205, gme3615, gme3759, gme3869, gme3905, gme4092*

Transcription factor jumonji	*gme1077, gme2932*
YL1 nuclear protein	*gme2893*
ssDNA-binding transcriptional regulator	*gme3272*
Zinc finger, MIZ-type	*gme218*
AT-rich interaction region	*gme129, gme2655*
CCR4-Not complex component, Not1	*gme1388*
Helix-turn-helix type 3	*gme1648*
Homeodomain-like	*gme2097, gme2358, gme2516, gme3303*
Zinc finger, DHHC-type	*gme226, gme2312, gme2378, gme4342*
Zinc finger, CCHC-type	*gme654, gme1985*
Transcription factor TFIIS	*gme2522, gme3819*
Bromodomain transcription factor	*gme3145, gme3608*

Bold to indicate knocked-out genes.

Supplementary Table S3. Peak area of main SLs compositions

Peak	RT (min)	SL structure	wt Area (10^6 mAU*s)	Δrlp Area (10^6 mAU*s)	ΔrlpΔleu3 Area (10^6 mAU*s)	ΔΔΔ Area (10^6 mAU*s)
1	9.20	C18:2 NASL	4.18±0.05	3.89±0.43	4.27±0.95	5.07±0.07
2	11.23	C18:1 MASL	3.30±0.11	3.52±0.22	4.12±1.74	5.59±0.02
3	14.86	C18:2 DASL	1.19±0.04	5.20±0.30	8.22±1.54	8.87±0.29
4	22.45	C18:2 DLSL	24.30±0.26	23.88±2.78	23.49±1.56	24.63±0.34
5	25.61	C18:1 DLSL	10.29±0.32	10.62±0.73	11.10±0.75	11.56±0.14

NASL non-acetylated acidic sophorolipid, MASL mono-acetylated acidic sophorolipid, DASL di-acetylated acidic sophorolipid, DLSL di-acetylated lactonic sophorolipid.
